# The association of long-term blood pressure variability with hemodialysis access thrombosis

**DOI:** 10.3389/fcvm.2022.881454

**Published:** 2022-08-04

**Authors:** Mu-Yang Hsieh, Chi-Hung Cheng, Chiu-Hui Chen, Min-Tsun Liao, Chih-Ching Lin, Ten-Fang Yang, Shao-Yuan Chuang, Chih-Cheng Wu

**Affiliations:** ^1^Division of Cardiology, Department of Internal Medicine, National Taiwan University Hospital Hsinchu Branch, Hsinchu, Taiwan; ^2^College of Medicine, National Taiwan University, Taipei, Taiwan; ^3^Department of Biological Science and Technology, National Yang Ming Chiao Tung University, Hsinchu, Taiwan; ^4^ANSN Clinic, Hsinchu, Taiwan; ^5^Hemodialysis Center, National Taiwan University Hospital Hsinchu Branch, Hsinchu, Taiwan; ^6^School of Medicine, National Yang Ming Chiao Tung University, Hsinchu, Taiwan; ^7^School of Medicine, National Yang-Ming University, Taipei, Taiwan; ^8^Division of Nephrology, Department of Medicine, Taipei Veterans General Hospital, Taipei, Taiwan; ^9^Institute of Population Health Science, National Health Research Institutes, Miaoli County, Taiwan; ^10^Center of Quality Management, National Taiwan University Hospital Hsinchu Branch, Hsinchu, Taiwan; ^11^Institute of Biomedical Engineering, National Tsing Hua University, Hsinchu, Taiwan; ^12^Institute of Cellular and System Medicine, National Health Research Institutes, Miaoli County, Taiwan

**Keywords:** blood pressure variability, hemodialysis, vascular access, thrombosis, stenosis, blood pressure, follow-up studies, renal dialysis

## Abstract

**Background:**

Blood pressure variability (BPV) is an important risk factor for cardiovascular events in hemodialysis patients. We sought to determine the impact of BPV on hemodialysis access thrombosis.

**Methods:**

We enrolled 1,011 prevalent hemodialysis patients from 12 hemodialysis centers since January 2018 and followed them until December 2020. Predialysis blood pressure (BP) was assessed at 12-week intervals. The coefficient of variation derived from 36 consecutive BP measurements was used as the metric for variability. The primary outcome was incident hemodialysis access thrombosis. Linear regression models were used to assess factors associated with BPV at baseline. Kaplan-Meier curves of the time until vascular access events were drawn and log-rank tests were calculated. Cox proportional hazards models were performed to assess the association of BPV with incident vascular access events.

**Results:**

The average coefficient of variance for systolic BPV was 10.9%. BPV was associated with age, body mass index, mean BP, diabetes, coronary and peripheral artery disease, history of access dysfunction, graft access, intradialytic hypotension, and use of antihypertensive medications. There were 194 access thrombosis events and 451 access stenosis events during a median follow-up period of 30 months. After adjustment of potential confounding factors, BPV was associated with increased risk of access thrombosis [hazard ratio = 1.27, 95% confidence interval (CI), 1.18–1.44, per 1 standard deviation increase in BPV]. The patients in the highest BPV quartile had 2.45 times the risk of thrombosis (CI, 1.62–3.70). The association was independent of average BP, intradialytic hypotension, and comorbidities. Similar trends of association were found in the subgroups analyzed. Comparative analysis using a time-varying variable model and different metrics of BPV showed consistent results.

**Conclusion:**

Our findings underscored the impact of BP fluctuation on vascular access thrombosis.

## Introduction

Hemodialysis access dysfunction continues to be a major source of morbidity and mortality in patients with end-stage renal disease (1). Thrombosis is the most common cause of access failure and also causes substantial stress on hemodialysis patients and their caregivers. Although stenosis is the most common cause of access thrombosis, 20–40% of thrombotic events occur in the absence of stenosis (2, 3). Moreover, various factors may precipitate the occurrence of thrombosis in an access with underlying stenosis (4). Factors other than stenosis are less well studied, but identifying these factors is critical for the prevention of dialysis access thrombosis.

Hypertension is a well-known risk factor for vascular diseases in the general population. Nonetheless, most studies found the association of vascular access thrombosis with hypotension (5–7). Blood pressure (BP) is the primary driving force of blood flow in the vascular access, and a low-flow state is one of the major determinants for thrombosis (8). Although this causal relationship makes sense, studies have rarely examined the relationship between BP and access thrombosis in a comprehensive manner. Most previous studies used average BP to investigate the relationship between BP and thrombosis. However, flow-related thrombosis should be susceptible to BP fluctuation as well, not only the usual BP. Hemodialysis patients are particularly prone to wide BP fluctuations, either due to comorbidities or unique dialysis factors. Although average BP is traditionally a risk factor for systemic vascular events, growing evidence suggests that BP instability and variability may contribute to the development of vascular events as well (9–11).

Both short-term (intradialytic or interdialytic period) or long-term (day-by-day or visit-by-visit) BP variability (BPV) are informative measures to predict vascular events (12). The effect of short-term BPV on the prognosis of hemodialysis patients has been reported. Intradialytic hypotension and intradialytic BP variability were associated with vascular access outcomes (6, 13, 14). Long-term BPV is reproducible over time but is only modestly related to short-term variability (15, 16). Growing evidence shows that long-term BPV predicts mortality and cardiovascular events in hemodialysis patients (9–11, 17–21). Nonetheless, the impact of long-term BPV on dialysis access remained unknown. Long-term BPV measurements are more feasible for hemodialysis patients than non-dialysis patients, which could be derived from BPs recorded during dialysis sessions.

We hypothesized that higher visit-to-visit BPV would increase the risk of vascular access thrombosis. Therefore, we conducted a prospective study to investigate the relation between BPV and dialysis access thrombosis.

## Materials and methods

### Study participants and design

**FIGURE 1:**
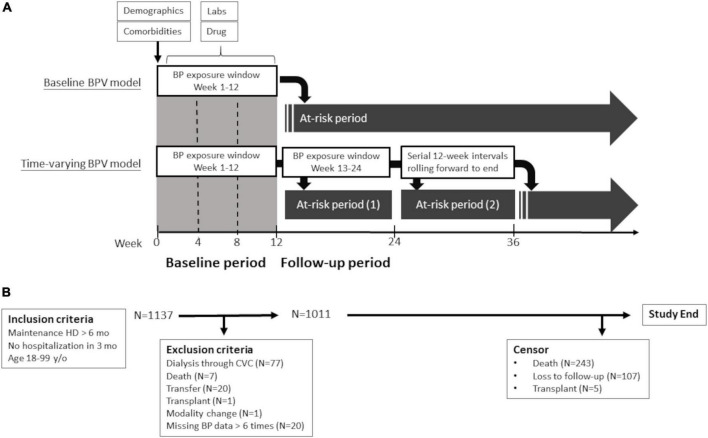
Study design **(A)** and flow diagram **(B)**. Flow diagram of study participants and schematic presentation of outcomes models for primary analysis (baseline BPV model) and comparative analysis (time-varying BPV model). BP, blood pressure; BPV, blood pressure variability; CVC, central vein catheter; HD, hemodialysis; and lab, laboratory data.

This is a prospective multicenter study (Hsinchu V.A. study, ClinicalTrials.gov identifier: NCT04692636) designed to investigate clinical factors related to cardiovascular events of maintenance hemodialysis patients. The study participants were recruited from January 1, 2018 to December 31, 2018, from 12 hemodialysis centers in the Hsinchu district of Taiwan. The Hsinchu district is composed of 1 city and 13 townships, with a total population of about 1,000,000. Four of the 12 hemodialysis centers are hospital-based centers and eight of them are dialysis clinics. The inclusion criteria were as follows: (1) age between 18 and 90 years old, (2) maintenance dialysis of more than 6 months, and (3) no hospitalization during the previous 3 months. For the analysis of hemodialysis access outcomes in this study, the following exclusion criteria were used during the exposure period: (1) use of central venous catheters in dialysis, (2) death, facility transfer, or modality change, (3) missing dialysis session, and (4) missing BP data > 10% (Figure 1).

We obtained information on patients’ demographics, comorbidities, dialysis-related information, medications, and laboratory data from the medical records of dialysis centers. These data were collected at baseline and updated every 3 months by trained study coordinators. BP data were extracted from a computerized dialysis information system (INDAS, Integrative Data Acquisition System, TW) in which vital signs and dialysis parameters from each dialysis session are stored digitally. Dialysis accesses were followed at all respective hemodialysis centers under the same protocol, and were referred to our angiographic unit for evaluation or management under the same criteria. Events of dialysis access were collected by reviewing the dialysis records, and angiography or surgery reports at a 3-month interval. Subjects were followed until December 31, 2020, and censored at the time of death, kidney transplant, or transfer to a non-study center. The study was approved by the Institutional Review Board and all patients signed an informed consent to participate in the study.

### Exposure variables

Blood pressure measurements were conducted before (pre-dialysis), during, and after (post-dialysis) each hemodialysis session, in a seated position, using automated oscillometric devices as per the dialysis unit routine. BP values were extracted from a computerized database every 90 days. We calculated the baseline mean BP and BPV using all the pre-dialysis BP measurements from week 1 to the end of week 12. During the follow-up period, we assessed the mean BP and BPV every 12 weeks. Allowable ranges were 50–300 mm Hg for systolic BP and 10 to 150 mm Hg for diastolic BP. When a value was outside the range, the data point was coded as missing. We only analyzed patients with BP data of more than 30 occasions to maintain excellent reproducibility of BPV data (Supplementary Figure 1). The BPV was calculated over a 12-week interval as the exposure for the next 12-week period at risk (Figure 1). The raw BP data were transformed into four BPV metrics: standard deviation (SD), coefficient of variance (CV), absolute real variation (ARV), and variance independent of the mean (VIM). ARV is calculated as the average absolute difference between consecutive measurements. VIM is a transformation of SD and is by definition uncorrelated to mean BP. VIM is calculated by fitting a curve through a plot of SD systolic BP (*y*-axis) against mean systolic BP (*x*-axis) with the parameter d estimated from the curve [VIM = (SD/mean*^x^*)] (22). We used the CV of consecutive 36 predialysis systolic BPs in the 12-week exposure period as the primary measure of BPV for analysis. We also used SD, VIM, and ARV of the predialysis BPs in the exposure period of the additional analysis.

### Covariates

The following domains of covariates were assessed over the first 12 weeks as the baseline level and updated every 12 weeks: demographic and anthropometric, clinical, dialysis (dialysis vintage, vascular access age, and vascular access type), laboratory, and medication. Demographic and anthropometric domains include age, sex, and body mass index. Clinical domains included diabetes, coronary artery disease, cerebrovascular disease, heart failure, and history of vascular access dysfunction. To account for other comorbidities, a comorbidity index previously validated in dialysis patients was calculated (23). Dialysis domains included dialysis vintage, shunt age, vascular access type (fistula vs. graft), urea clearance, relative fluid removed per dialysis session [(pre-dialysis weight-post-dialysis weight)/post-dialysis weight], and dialysis institutions (dialysis clinics or hospital-based dialysis center). Laboratory domains included serum albumin, cholesterol, hemoglobin, calcium, and phosphate product were collected every 12 weeks. Medication domains included antiplatelet, anticoagulant, statin, antihypertensive medicines. We also included mean predialysis SBP and intradialytic hypotension as covariates. Intradialytic hypotension was defined as a nadir SBP less than 90 mm Hg in at least 30% of dialysis sessions (24).

### Outcome measurements

The surveillance protocol of dialysis access included physical examination, dynamic venous pressure monitoring at each dialysis session and dialysis dose, transonic examination of access flow (if available) at monthly interval. Patients were referred to our angiographic unit for evaluation on the basis of the following criteria: (1) clinical manifestations suggesting vascular access dysfunction (decreased thrill, abnormal bruit, increased pulsatility, and prolonged bleeding from the puncture site); (2) a reduction in flow rate of >25% from baseline; (3) total access blood flow <500 ml/min by ultrasound dilution method; and (4) increased venous pressure during dialysis (dynamic venous pressure exceeding threshold level measured three consecutive times); and (5) unexplained decrease (>0.2 unit) in the delivered dialysis dose. Our primary outcome of interest was the time to the first vascular access thrombosis, defined as the access that had clotted without blood flow. Information of vascular access thrombosis was obtained from documentation in the dialysis records, angiography reports, or surgery reports. The secondary outcome was the time to the first vascular access stenosis, defined as the anatomical stenosis of more than 50% by angiography with corresponding clinical or hemodynamic abnormalities of the referring criteria. Because the patient may change vascular access throughout the follow-up period, the only outcome of first vascular access was used for analysis. In the primary baseline model analysis, vascular access outcomes were defined as the first event that occurred from week 13 to the end of the study or censoring. In the companion timing-varying analyses, vascular outcomes were defined as the first event within 12 weeks after the end of the 12-week exposure period (Figure 1).

### Statistical analysis

We reported patients’ baseline characteristics across quantiles of systolic BPV. We assessed factors associated with BPV at baseline using linear regression models. Kaplan-Meier curves of the time until vascular access events were drawn and log-rank tests were calculated. Cox proportional hazards models were performed to assess the association of BPV with incident vascular access events. Three sets of analyses were examined to access the relationship between BPV and vascular access events. The primary analysis describes the relationship between baseline BPV (derived from a 12-week exposure period) and vascular access outcomes. The first sensitivity analysis used time-varying BPV (calculated over a 12-week interval) as well as other time-varying covariates for analysis. The second sensitivity analysis excluded thrombosis or stenosis events during hospitalization in order to eliminate the hemodynamic effect due to acute illness. Univariate analyses were done before proceeding to multivariate models. Unadjusted and adjusted models with hazard ratios (HRs) and 95% CI were presented per 1-SD increase of BPV. Age, sex, and mean SBP were considered as potential *a priori* confounders. Significance thresholds of 0.1 in the univariate linear regression model were selected as potential confounding factors in multivariate models. Proportional hazard assumption was checked graphically using the log-log plot and was found to be acceptable for the factors of interest. Subgroup analyses were performed on patients defined by age, sex, systolic BP, diabetes, coronary artery disease, intradialytic hypotension, vascular access, and institution. These interaction of these factors on the associations between BPV and thrombosis events were assessed by adding interaction terms in the model. We considered two-tailed *P* values < 0.05 as statistically significant. All analyses were performed using the SPSS software (IBM SPSS Statistics for Windows, Version 22.0. Armonk, NY, United States: IBM Corp).

## Results

### Baseline characteristics

A total of 1,137 patients were enrolled. After the exclusion of 77 patients dialyzed *via* central vein catheters and 49 patients for other reasons, the final cohort included for analysis consisted of 1,011 patients (Figure 1). Baseline characteristics of the study population are presented in Table 1. The mean age of the study participants was 66 years (SD of 14 years) and 521 of them were male (52%). Their median hemodialysis duration was 59 months, median shunt age was 56 months, and 192 patients (19%) using arteriovenous graft (AVG) as their regular vascular access at enrollment. Baseline characteristics of the final cohort were similar to the hemodialysis patients of a nationwide survey in 2017 (Supplementary Table 1). These patients were followed up for a median of 30 months and 243 patients died during the follow-up period. Five patients received a kidney transplant and 107 patients were lost to follow-up or transferred to non-study centers. A total of 451 patients had vascular access stenosis events and 194 patients had vascular access thrombosis events.

**TABLE 1:** Baseline characteristics stratified by systolic BPV quartiles.

Factors	Overall (*N* = 1,011)	BPV quartiles			
		Q1 (*N* = 253)	Q2 (*N* = 253)	Q3 (*N* = 253)	Q4 (*N* = 252)
**Demographics**					
Age, year	66 ± 14	65 ± 14	65 ± 14	66 ± 14	68 ± 15
Sex,% male	521 (52)	139 (60)	132 (52)	125 (49)	125 (50)
Body mass index	22.9 ± 4.3	22.2 ± 3.7	22.4 ± 4.2	23.3 ± 4.0	23.7 ± 5.0
<18.5	129 (13)	34 (13)	42 (17)	21 (8)	32 (13)
18.5–24	523 (52)	147 (58)	131 (52)	132 (52)	113 (45)
≥24	359 (36)	72 (28)	80 (32)	100 (40)	107 (42)
**Blood pressure**					
Systolic BP, mm Hg	141 ± 23	136 ± 24	140 ± 25	142 ± 23	144 ± 20
Diastolic BP, mm Hg	71 ± 12	69 ± 12	69 ± 13	72 ± 12	74 ± 12
Systolic BPV,%	10.9 ± 3.0	7.7 ± 0.9	9.7 ± 0.5	11.4 ± 0.6	14.9 ± 2.4
**Clinical history**					
Smoking (%)	139 (14)	28 (11)	46 (18)	28 (11)	37 (15)
Diabetes (%)	555 (55)	124 (49)	129 (51)	136 (54)	166 (66)
CAD (%)	350 (35)	71 (28)	89 (35)	104 (41)	86 (34)
PAD (%)	75 (7)	12 (5)	18 (7)	21 (8)	24 (10)
CHF (%)	150 (15)	36 (14)	42 (17)	35 (14)	37 (15)
CVA (%)	90 (9)	20 (8)	19 (8)	26 (10)	25 (10)
History of VAD (%)	270 (27)	60 (24)	66 (26)	67 (27)	78 (31)
Comorbidity index	4.80 (1.45)	4.58 (1.66)	4.68 (1.67)	5.01 (1.68)	4.92 (1.68)
**HD-related**					
Duration, month	59 ± 68	62 ± 68	56 ± 67	59 ± 71	58 ± 64
Shunt age, month	57 ± 66	61 ± 67	55 ± 66	53 ± 65	55 ± 64
Frequency/week	2.94 ± 0.25	2.94 ± 0.26	2.93 ± 0.27	2.93 ± 0.27	2.96 ± 0.2
Kt/V	1.39 ± 0.23	1.41 ± 0.21	1.38 ± 0.24	1.38 ± 0.2	1.39 ± 0.24
Fluid removal,%	3.58 ± 1.44	3.59 ± 1.38	3.61 ± 1.46	3.55 ± 1.34	3.58 ± 1.59
IDH,%	289 (28)	74 (29)	66 (26)	60 (22)	89 (35)
Hospital HD,%	554 (55)	153 (60)	132 (52)	133 (47)	136 (46)
Access (AVG,%)	192 (19)	43 (17)	38 (15)	55 (22)	56 (22)
**Laboratory**					
Albumin, g/dl	3.8 ± 0.4	3.8 ± 0.4	3.8 ± 0.4	3.8 ± 0.4	3.7 ± 0.4
Cholesterol, mg/dl	161 ± 38	165 ± 36	160 ± 37	159 ± 40	160 ± 38
Ca X P	46 ± 14	47 ± 13	46 ± 15	45 ± 14	46 ± 14
Hb, g/dl	10.7 ± 1.5	10.8 ± 1.5	10.7 ± 1.4	10.5 ± 1.5	10.7 ± 1.4
**Medication**					
Antiplatelet (%)	239 (24)	47 (19)	61 (24)	75 (30)	56 (22)
Single agent (%)	188 (64)	39 (83)	48 (79)	61 (81)	40 (71)
Dual agents (%)	51 (36)	8 (17)	13 (21)	14 (19)	16 (29)
VKA/NOAC (%)	11 (1.1)	2 (0.8)	5 (2.0)	1 (0.4)	3 (1.2)
Statin (%)	144 (14)	31 (12)	36 (14)	38 (15)	39 (15)
Anti-hypertension (%)	416 (41)	141 (56)	90 (36)	98 (39)	87 (35)
Beta-blocker (%)	196 (47)	83 (59)	47 (52)	52 (53)	38 (44)
RAS inhibitor (%)	164 (39)	53 (38)	38 (42)	42 (43)	31 (36)
Calcium blocker (%)	241 (58)	65 (46)	71 (79)	53 (54)	52 (60)
Others (%)	88 (21)	23 (16)	23 (26)	24 (24)	18 (21)

AVG, arteriovenous graft; BP, blood pressure; BPV, blood pressure variability, by coefficient variation; CAD, coronary artery disease, Ca x P, calcium phosphate product; CHF, congestive heart failure; CVA, cerebrovascular accident; Hb, hemoglobin; HD, hemodialysis; IDH, intradialytic hypotension; KT/V, urea clearance; NOAC, novel oral anticoagulant; PAD, peripheral artery disease; Q, quartile; RAS, renin-angiotensin system; VAD, vascular access dysfunction; and VKA, vitamin-K antagonist.

### Blood pressure and blood pressure variability metrics

The mean pre-dialysis systolic BP was 141 mm Hg and diastolic BP was 71 mm Hg. The average CV for systolic BP was 10.9% (SD, 3.0%). Other metrics of BPV, such as SD, ARV, and VIM, were provided in Supplementary Table 2. Table 2 shows that baseline BPV was correlated with age, body mass index, systolic BP, diabetes, coronary artery disease, peripheral artery disease, vascular access dysfunction, comorbidity index, AVG, intradialytic hypotension, albumin level, cholesterol level, and use of antihypertensive medications.

**TABLE 2:** Factors correlated with Systolic BPV.

Factors (unit)	β	(SE)	*P* value
**Demographics**			
Age (year)	0.01	0.01	0.03
Sex (women vs. men)	–0.19	0.19	0.32
Body mass index (1)	0.09	0.02	< 0.001
Systolic blood pressure (1 mm Hg)	0.02	0.01	< 0.001
**Clinical**			
Smoking (vs. no)	0.08	0.27	0.76
Diabetes (vs. no)	0.81	0.19	< 0.001
History of CAD (vs. no)	0.44	0.19	0.03
History of PAD (vs. no)	1.08	0.35	0.002
History of CVA (vs. no)	0.46	0.33	0.16
History of VAD (vs. no)	0.45	0.21	0.03
Comorbidity index (1)	0.18	0.06	0.001
**HD-related**			
Kt/V (1)	–0.39	0.41	0.34
Fluid removal (1%)	–0.05	0.06	0.47
Hospital HD (vs. clinic)	–0.26	0.19	0.16
AVG (vs. AVF)	0.60	0.24	0.01
IDH (vs. no)	0.41	0.21	0.05
**Laboratory data**			
Albumin (1 g/dl)	–0.645	0.243	0.008
Hemoglobin (1 g/dl)	–0.06	0.06	0.31
**Medication**			
Antiplatelet (vs. no)	0.12	0.22	0.58
VKA/NOAC (vs. no)	0.34	0.89	0.70
Antihypertensive (vs. no)	–0.45	0.19	0.02
Beta-blocker (vs. no)	–0.48	0.29	0.09
RAS inhibitor (vs. no)	–0.09	0.30	0.77
Calcium blocker (vs. no)	0.34	0.29	0.25

AVF, arteriovenous fistula; AVG, arteriovenous graft; BPV, blood pressure variability, by coefficient variation; Ca x P, calcium phosphate product; CAD, coronary artery disease, CHF, congestive heart failure; CVA, cerebrovascular accident; Hb, hemoglobin; HD, hemodialysis, VKA, vitamin-K antagonist, NOAC, novel oral anticoagulant; IDH, intradialysis hypotension; KT/V, urea clearance; and VAD, vascular access dysfunction.

### Effect of systolic blood pressure and blood pressure variability on outcomes of vascular access

**FIGURE 2:**
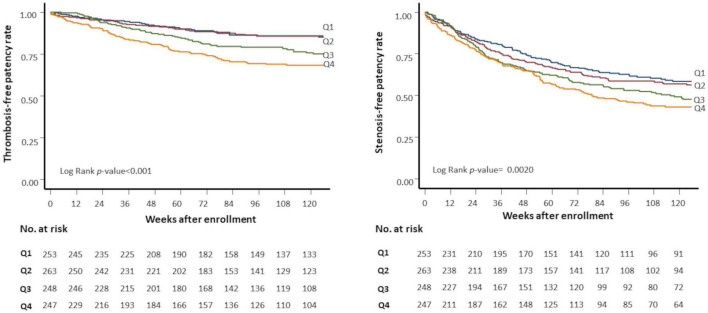
Kaplan-Meier plots of vascular access outcomes. Kaplan-Meier plots of stenosis-free patency (left panel) and thrombosis-free patency (right panel) rates by quartiles of predialysis systolic blood pressure variability (SBPV). Q1 (blue line), first (lowest) SBPV quartile; Q2 (red line), second quartile; Q3 (green line), third quartile; Q4 (yellow line), fourth (highest) SBPV quartile. No. at risk, the number of patients at risk.

**FIGURE 3:**
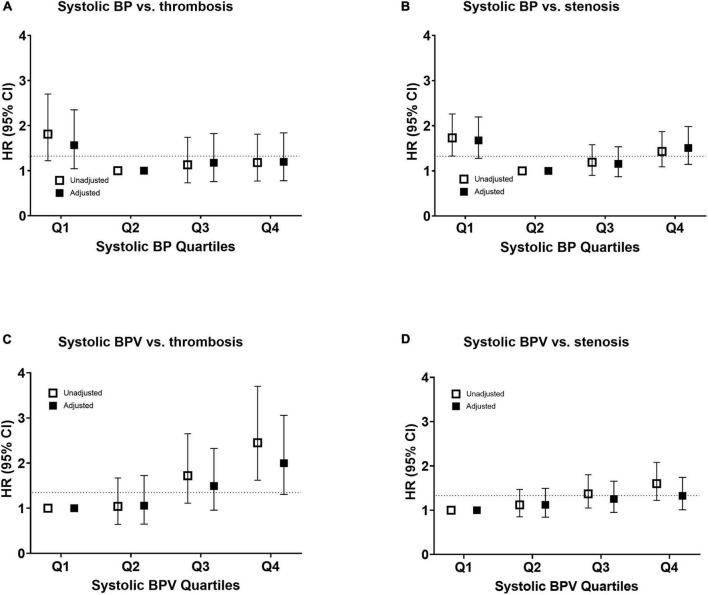
Risk of vascular access outcomes by systolic blood pressure and variability. Unadjusted and adjusted hazard ratio of thrombosis **(A,C)** and stenosis **(B,D)** events by quantiles of mean systolic blood pressure in panels **(A,B)** and systolic blood pressure variability in panels **(C,D)**. The risk was adjusted for age, sex, and all the baseline factors with a *p*-value < 0.1 in the univariate Cox regression analysis. The squares indicate the hazard ratio and the error bars indicate 95% confidence intervals. CI, confidence interval; HR, hazard ratio; SBP, systolic blood pressure; and SBPV, systolic blood pressure variability.

Baseline mean BP was not associated with stenosis or thrombosis of dialysis access. When the mean BP was stratified into quartiles, the lowest BP group (quartile 1) had a higher risk of access thrombosis (Table 3). The BP quartiles were not associated with vascular access stenosis. Baseline BPV was associated with vascular access stenosis (Figure 2). In univariate Cox regression analysis, per 1 SD increase of BPV was associated with a 17% increased risk of stenosis [HR, 1.17; confidence interval (CI), 1.08–1.28, *P* < 0.001]. When BPV was stratified into quartiles, the highest BPV quartile had 1.60 times (CI, 1.22–2.08, *P* < 0.001) risk of stenosis than the lowest quartile (Table 3). After multivariate adjustment, BPV did not associate with risk of stenosis (Table 4). Baseline BPV was associated with thrombosis of vascular access (Figure 2). In univariate Cox regression analysis, every 1 SD increase in BPV was associated with a 35.7% increased risk of thrombosis (HR, 1.36; CI, 1.21–1.52, *P* < 0.001). When BPV was stratified into quartiles, the highest BPV quintile had 2.45 times the risk of thrombosis (1.62–3.70, *P* < 0.001) than the lowest quartile, with a graded effect among the quartiles (Table 3). After multivariate adjustment, BPV remained significantly associated with the risk of thrombosis (Table 4). The relationship between BP and BPV quartiles with vascular access outcomes is displayed in Figure 3.

**TABLE 3:** Univariate Cox regression analysis of predictors for incident vascular access thrombosis or stenosis during follow-up period.

Variables (Unit of increase)	Thrombosis				Stenosis			
	HR	95% CI		*P* value	HR	95% CI		*P* value
**Demographics**								
Age (1 year)	1.00	0.99	1.01	0.70	1.00	0.99	1.01	0.84
Male sex (yes)	0.81	0.61	1.08	0.15	0.90	0.75	1.08	0.27
BMI (1)	1.00	0.97	1.04	0.98	1.01	0.99	1.04	0.28
**Clinical**								
Smoking (yes)	1.12	0.76	1.65	0.58				
Diabetes (yes)	1.40	1.05	1.87	0.02	1.15	0.95	1.38	0.16
History of CAD (yes)	1.46	1.10	1.93	0.009	1.05	0.86	1.27	0.66
History of PAD (yes)	1.77	1.14	2.73	0.01	1.75	1.29	2.37	< 0.01
History of CHF (yes)	0.85	0.56	1.28	0.43	0.94	0.72	1.22	0.64
History of CVA (yes)	1.10	0.69	1.77	0.69	1.12	0.82	1.53	0.48
History of VAD (yes)	2.75	2.01	3.60	< 0.001	2.76	2.29	3.33	< 0.001
Comorbidity index (1)	1.13	1.04	1.22	0.003	1.04	0.99	1.10	0.15
**HD-related**								
HD duration (1 month)	1.00	1.00	1.00	0.94	1.00	1.00	1.00	0.16
Shunt age (1 month)	1.00	1.00	1.00	0.30	1.00	1.00	1.00	0.17
Kt/V (1)	2.42	1.28	4.54	0.006	1.50	0.98	2.30	0.07
Fluid removal (1%)*^a^*	1.05	0.96	1.16	0.29	1.04	0.98	1.11	0.22
IDH (yes)	2.19	1.65	2.92	< 0.001	2.02	1.68	2.44	< 0.001
Hospital HD (yes)	0.68	0.51	0.90	0.007	0.79	0.66	0.95	0.01
AVG (yes)	5.37	4.04	7.13	< 0.001	2.32	1.89	2.86	< 0.001
**Laboratory data**								
Albumin (1 g/dl)	0.75	0.52	1.07	0.11	1.08	0.85	1.37	0.55
Cholesterol (1 mg/dl)	1.00	1.00	1.00	0.89	1.00	1.00	1.00	0.62
Ca X P (1)	0.99	0.98	1.00	0.26	1.00	0.99	1.00	0.16
Hb (1 g/dl)	1.00	0.90	1.10	0.92	0.98	0.92	1.05	0.56
**Medication**								
Antiplatelet (yes)	1.21	0.88	1.66	0.23	1.19	0.97	1.47	0.10
Dual agents (yes)	0.97	0.51	1.84	0.93	0.93	0.61	1.43	0.74
VKA/NOAC (yes)	2.29	0.85	6.18	0.10	1.61	0.76	3.40	0.21
Statin (yes)	1.25	0.86	1.82	0.24	1.30	1.01	1.66	0.04
Anti-HT (yes)	0.75	0.55	1.01	0.05	0.79	0.65	0.96	0.02
Beta-blocker (yes)	0.79	0.49	1.30	0.34	0.99	0.73	0.13	0.95
RAS inhibitor (yes)	0.84	0.51	1.39	0.50	0.80	0.58	1.10	0.17
Calcium blocker (yes)	0.89	0.55	1.43	0.62	0.86	0.64	1.17	0.35
**Systolic BP**								
Quartile 1	1.81	1.22	2.70	0.003	1.73	1.33	2.26	< 0.001
Quartile 2	Ref				Ref			
Quartile 3	1.13	0.73	1.74	0.59	1.19	0.90	1.58	0.22
Quartile 4	1.18	0.77	1.81	0.45	1.43	1.09	1.87	0.01
**Systolic BPV**								
Quartile 1	Ref				Ref			
Quartile 2	1.04	0.64	1.67	0.88	1.12	0.85	1.47	0.43
Quartile 3	1.72	1.11	2.65	0.01	1.37	1.05	1.80	0.02
Quartile 4	2.45	1.62	3.70	< 0.001	1.60	1.22	2.08	< 0.001

AVF, arteriovenous fistula; AVG, arteriovenous graft; BMI, body mass index; BP, blood pressure; BPV, blood pressure variability, by coefficient variation; Ca x P, calcium phosphate product; CAD, coronary artery disease, CHF, congestive heart failure; CVA, cerebrovascular accident; Hb, hemoglobin; HD, hemodialysis, IDH, intradialysis hypotension; Kt/V, urea clearance; NOAC, novel oral anticoagulant; PAD, peripheral artery disease; VAD, vascular access dysfunction; VKA, vitamin-K antagonist; Quartile 1, lowest quartile; and Quartile 4, highest quartile.

^a^ Per 1% post-dialysis body weight.

**TABLE 4:** Multivariate Cox regression analysis of the association between BPV and outcomes of vascular access, displayed by the whole cohort and stratified by access types.

Access type	Thrombosis				Stenosis			
	HR	95% CI (LB)	95% CI (UB)	*P* value	HR	95% CI (LB)	95% CI (UB)	*P* value
**Fistula + Graft**								
Crude	1.36	1.21	1.52	< 0.001	1.17	1.08	1.28	< 0.001
Model 1	1.34	1.19	1.51	< 0.001	1.16	1.07	1.27	0.001
Model 2	1.26	1.11	1.43	< 0.001	1.08	0.99	1.18	0.07
Model 3	1.26	1.11	1.43	< 0.001	1.09	0.99	1.19	0.07
**Fistula**								
Crude	1.33	1.13	1.56	< 0.001	1.15	1.04	1.27	0.009
Model 1	1.31	1.11	1.54	0.001	1.14	1.03	1.26	0.01
Model 2	1.20	1.02	1.42	0.03	1.07	0.97	1.19	0.18
Model 3	1.20	1.01	1.41	0.04	1.07	0.96	1.19	0.23
**Graft**								
Crude	1.33	1.11	1.59	0.002	1.16	0.99	1.37	0.07
Model 1	1.40	1.16	1.69	< 0.001	1.19	1.01	1.41	0.04
Model 2	1.34	1.09	1.65	0.006	1.14	0.95	1.36	0.17
Model 3	1.40	1.13	1.73	0.002	1.17	0.98	1.41	0.09

CI, confidence interval; HR, hazard ratio; LB, lower bound; and UB, upper bound.

Model 1: adjusted for age, sex, systolic blood pressure.

Model 2: adjusted for factors with p value less than 0.1 in univariate analyses.

For analysis of thrombosis, including diabetes, coronary artery disease, peripheral artery disease, vascular access dysfunction, Charlson comorbidity index, urea clearance, intradialysis hypotension, institution, access types, and systolic blood pressure.

For analysis of stenosis, including Charlson comorbidity index, peripheral artery disease, vascular access dysfunction, urea clearance, intradialysis hypotension, institution, type of access, anti-hypertension medicine, and statin.

Model 3: all factors in the model 1 and model 2.

### Subgroup analyses

**FIGURE 4:**
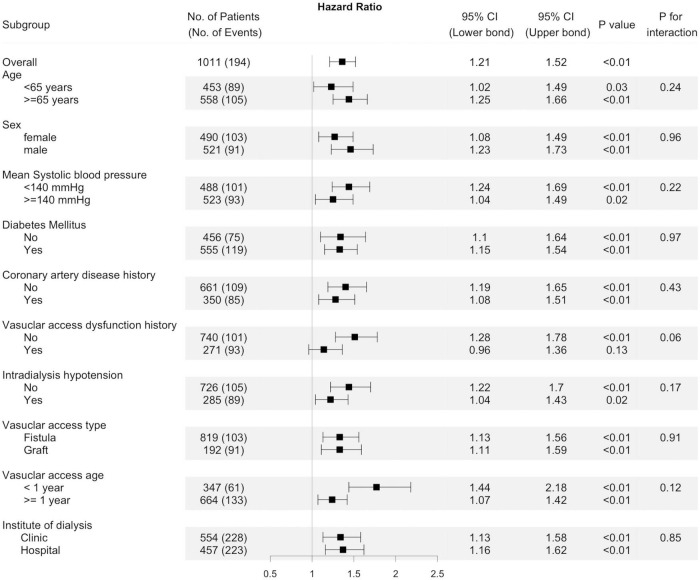
Subgroup analyses. Unadjusted hazard ratio of thrombosis events per one standard deviation increase of predialysis systolic blood pressure variability (BPV) in different subgroups of patients. The interaction of factors in each subgroup on the association of BPV with thrombosis were presented by the “*p* for interaction.”

Because the risk of events differed significantly between arteriovenous fistula (AVF) and AVG, we analyzed the association of BPV and thrombosis by access types (Table 4). After adjustment, BPV remains associated with thrombosis both in patients with AVF and AVG. The results in the univariable model were stratified by potential confounders, shown in Figure 4. Subgroup analysis showed that the association between BPV and thrombosis remained significant in most subgroups, except for patients with history of vascular access dysfunction.

### Sensitivity analysis

To account for the change of BPV over time, we analyzed the association of BPV and dialysis access outcomes by treating BPV as a time-dependent variable, rolling over 12 weeks. The effect of BPV on vascular access outcomes, regardless of stenosis or thrombosis, was similar to that observed in the baseline BPV model (Supplementary Table 3). To account for the effect of mean BP, different metrics of BPV, such as VIM and ARV, were used in the analysis. Analysis of these BP-independent indices with vascular access outcomes showed similar results, both for stenosis events and thrombosis events (Supplementary Table 4). After excluding 5 stenosis events and 4 thrombosis events during hospitalization, the effect of systolic BPV on vascular outcomes was similar to that in the primary analysis (Supplementary Table 5).

## Discussion

Visit-to-visit BPV is emerging as a relevant risk factor for cardiovascular events in dialysis and non-dialysis patients (9, 11, 25, 26). We showed in this cohort that the average predialysis systolic BPV was 10.9%, high than 6.1% in the general populations and similar to 9.9–10.7% reported in hemodialysis patients (11, 27, 28). Our analysis shows for the first time that visit-to-visit BPV is significantly associated with dialysis access thrombosis, either fistulas or grafts. This association is independent of baseline BP levels or intradialytic hypotension. After the adjustment of several confounding factors, BPV remained a significant predictor of thrombosis. Each SD increase in BPV was associated with a 26.8% increased risk of thrombosis, and the highest BPV quartile (CV > 14.9%) had a 2.45 times higher risk of thrombosis. Thus, visit-to-visit BPV seems to be a potential predictor and a probable modifiable risk factor for thrombosis of vascular access.

A low-flow state is a critical determinant for access thrombosis. Arterial BP is the primary driving force of access blood flow. Low BP has been shown to decrease access flow and increase thrombosis events. In the absence of structural abnormalities, low BP accounted for at least 20–40% access thrombosis (5). In previous studies, mean BP was used to evaluate the effect of BP on the risk of thrombosis, with an increase from 3% to 11% per SD decrease of BP. Nonetheless, greater fluctuations and lower nadir of BP theoretically increase the exposure to low-flow state. We showed that the highest BPV quartile had a 2.45 times higher risk of thrombosis than the lowest quartile. In the subgroup analysis, the effect was more prominent among individuals with systolic BP < 140 mm Hg, suggesting a pathogenic link to low BP burden. Although BP and BPV were closely related, the effect of BPV remained even after meticulous adjustment by different methods. Taking the above evidence together, BP fluctuation is a relevant risk factor for vascular access thrombosis.

Previous studies of the relationship between BP and vascular access focused on short-term variability or instability during dialysis. For example, a secondary analysis of the Hemodialysis (HEMO) Study revealed frequent episodes of intradialytic hypotension associated with AV fistula thrombosis. Two recent retrospective studies also found that the variability of intradialytic BP can predict access thrombosis (13, 14). Nonetheless, in most circumstances, access thrombosis occurred in the inter-dialytic period. The multivariate and subgroup analysis in our analyses suggest that the predictive value of long-term BPV was independent of intradialytic hypotension. In addition to arterial stiffness, long-term variability was more affected by environmental and behavioral factors. In contrast, short-term variability during dialysis was susceptible to cardiac function, arterial stiffness, and dialysis factors (6, 29). Previous studies demonstrated that a greater interdialytic weight gain or ultrafiltration volume was associated with short-term variability during dialysis. In our study, long-term BPV was not affected by fluid removal or urea clearance, as was also demonstrated in previous studies (9, 11). Therefore, the predictive value of long-term BPV was independent and complementary to intradialytic variability (27, 30).

There were other possible reasons for the association between BPV and thrombosis. Baseline BPV was associated with risk factors of arterial stiffness, such as aging, obesity, diabetes, and vascular diseases (31). Diabetes and vascular diseases also increased the risk of access thrombosis. BPV may only be an intermediate factor in the thrombosis caused by diabetes and vascular diseases. Nonetheless, BPV predicted thrombosis after adjusting for diabetes and vascular diseases. The subgroup analysis also demonstrated that the association was independent of diabetes or vascular diseases. Therefore, BPV may not only be a marker of underlying vascular diseases but also a maker of thrombosis events. Dialysis access types are associated with BPV, as demonstrated in our results and previous studies (32). We analyzed the effect of BPV by access types because the anatomical configuration and hence hemodynamics of fistulas and grafts differ greatly. Fistulas require only one surgical anastomosis and are almost entirely endothelialized that do not often thrombose. Grafts are composed of unendothelialized foreign material that are inherently thrombogenic and had five-times risk of thrombosis in this study. Nonetheless, even after stratification by access types, high BPV remains a significant risk factor of thrombosis, either for fistulas or grafts.

Blood pressure variability was associated with stenosis of vascular access in univariate analysis. Intimal hyperplasia at the outflow vein is the most common cause of stenosis for either fistulas or grafts (33). BPV was reported to be associated with inflammation and endothelial dysfunction (31, 34), that may be the links to the development of intimal hyperplasia (35, 36). Arterial stiffness is one of the mechanisms for visit-to-visit BPV (37, 38). We indeed found that BPV was associated with risk factors of arterial stiffness, such as age, diabetes, and vascular diseases. Therefore, the association between BPV and stenosis may be mediated *via* these common pathogenic pathways as well (35, 36, 39). After multivariate adjustment, the risk estimates and significance of the association between BPV and stenosis decreased. Therefore, BPV is more likely to be a risk marker of stenosis rather than a risk maker.

Currently, no standardized method is available for assessing long-term BPV, either in dialysis or non-dialysis patients. The density of BP measurements varied widely among previous studies. In our study, high-density measurement (36 times in 12 weeks) was used to estimate BPV based on data from a digitized dialysis information system. Although such a high-density measurement is not easy for non-dialysis patients, it can be obtained from regular dialysis sessions without additional loadings on the staff. Home BP is addressed in recent guidelines for BPV assessment. Nonetheless, device standardization, measurement, timing, and data transformation are all potential obstacles to the application of home BP in clinical practice. In contrast, using clinic BP in dialysis centers is more feasible and the prognostic value was well-documented in previous studies (9–11). We used CV, which also accounted for mean BP as the primary BPV metric for analysis. Other BPV metrics, such as VIM and ARV, are available to reflect the fluctuation independent of average BP. Nonetheless, these metrics require complex calculations, which limits their clinical applicability.

Our study has several strengths. It was conducted in a typical clinical setting using a representative community-based cohort, with characteristics comparable to that of a nationwide registry. The detailed time-varying patient-level data on key variables were available, including fluid removal and anti-hypertensive medications, detailed ascertainment of comorbidities using medical records, and prospective ascertainment of vascular access events using dialysis records or angiographic reports. It is a multi-center study with a longitudinal study design and a long-term follow-up period, allowing us to collect repeated BP measurements, relevant confounders, and reliable outcomes. Unlike most studies that focused on a certain BPV indicator, we used several BPV indices to strengthen our findings. We used high-density measurements, which rendered a stable and reproducible BPV.

Some limitations should be addressed. Our sample was relatively small compared to nationwide registries or claims databases. From the research point of view, VIM is an ideal approach to exclude the influence of usual BP. However, in terms of clinical decision making, a CV may be a more practical option. Although the use of medications was adjusted, we did not have a direct assessment of medication adherence. BPV measurements were performed as part of routine clinical practice and we did not standardize the device for BP measurement. Different devices used for BP measurement could be a source of extra variability. The validity for lower-density BP measurements was not clear. A high-density BPV measurement is laborious and the assistance of information technology is important in clinical practice. We did not examine variability of diastolic BP or mean BP because studies on diastolic BPV are relatively few and have conflicting results (12, 28, 40). Finally, the relative few vascular thrombosis events may have contributed to the lack of a significant findings among the subgroups.

Given the high cost and prevalence of vascular access thrombosis, finding modifiable risk factors and implementable strategies may improve patient outcomes and healthcare burden. The current practice already dictates the association of BP variability and instability with mortality and cardiovascular events. Our results expanded the role of BPV in vascular access thrombosis events, either in fistulas or grafts. The impact of BPV is more pronounced in dialysis patients with normal BP. Uncertainty regarding appropriate BP targets in hemodialysis patients continues, and whether higher BP targets are warranted in patients at risk of access thrombosis deserved further investigation. Evidence derived from *post hoc* analyses of previous studies suggested that certain classes of antihypertensive medicines were more effective on BPV than others (41). Definite answers can only be provided by randomized clinical trials. Our analysis underscores the importance of including vascular access patency as a relevant outcome in future studies on BP management of hemodialysis patients. The importance of BP fluctuation on vascular access thrombosis should be emphasized, rather than focusing on average BP alone. Further studies are needed to assess whether maintaining the stability of BP can be a promising target in the prevention of vascular access thrombosis.

## Data availability statement

The raw data supporting the conclusions of this article will be made available by the authors, without undue reservation.

## Ethics statement

The studies involving human participants were reviewed and approved by National University Hospital, Hsinchu Branch, IRB. The patients/participants provided their written informed consent to participate in this study.

## Author contributions

M-YH and C-CW: research idea and study design. M-YH, S-YC, Chi-HC, and Chiu-HC: data acquisition. M-TL, S-YC, and C-CW: data analysis/interpretation. M-TL and S-YC: statistical analysis. C-CW: supervision or mentorship. All author contributed important intellectual content during manuscript drafting or revision and agrees to be personally accountable for the individual’s own contributions and to ensure that questions pertaining to the accuracy or integrity of any portion of the work, even one in which the author was not directly involved, are appropriately investigated and resolved, including with documentation in the literature if appropriate.
