# Supplementary material for: The association of long-term blood pressure variability with hemodialysis access thrombosis
Source: Front Cardiovasc Med. 2022 Aug 4;9:881454. doi: 10.3389/fcvm.2022.881454 (PMC9386040; doi:10.3389/fcvm.2022.881454)
Supplement: Supplementary file 1 [file Data_Sheet_1.docx]

**The association of long-term blood pressure variability with hemodialysis access thrombosis**

Mu-Yang Hsieh, MD^1,2,3^, Chi-Hung Cheng, MD^4^, Chiu-Hui Chen, RN^5^, Min-Tsun Liao, MD, PhD^1,2^, Chih-Ching Lin, MD, PhD^6,7,8^, Ten-Fang Yang, MD, MSc, PhD^3^, Shao-Yuan Chuang, PhD^9^, Chih-Cheng Wu, MD^2,10,11,12^

1. Division of Cardiology, Department of Internal Medicine, National Taiwan University Hospital Hsinchu Branch, Hsinchu, Taiwan
2. College of Medicine, National Taiwan University, Taipei, Taiwan
3. Department of Biological Science and Technology, National Yang Ming Chiao Tung University, Hsinchu, Taiwan.
4. ANSN Clinic, Hsinchu, Taiwan
5. Hemodialysis Center, National Taiwan University Hospital Hsinchu Branch, Hsinchu, Taiwan
6. School of Medicine, National Yang Ming Chiao Tung University, Hsinchu, Taiwan
7. School of Medicine, National Yang-Ming University, Taipei Taiwan
8. Division of Nephrology, Department of Medicine, Taipei Veterans General Hospital, Taipei, Taiwan
9. Institute of Population Health Science, National Health Research Institutes, Zhunan, Miaoli County, Taiwan
10. Center of Quality Management, National Taiwan University Hospital Hsinchu Branch, Hsinchu, Taiwan
11. Institute of Biomedical Engineering, National Tsing Hua University, Hsinchu, Taiwan
12. Institute of Cellular and System Medicine, National Health Research Institutes, Zhunan, Miaoli County, Taiwan

**Correspondence to:**

Chih-Cheng Wu, MD

Associate Professor, College of Medicine, National Taiwan University, Taipei, Taiwan;

Director, Quality Control Center, National Taiwan University Hospital Hsinchu Branch, Hsinchu, Taiwan

No. 25, Lane 442, Section 1, Jingguo Road, Hsinchu, Taiwan

Phone: +886-3-5326151 ext. 2010; E-mail: [chihchengwumd@gmail.com](mailto:chihchengwumd@gmail.com)

SY Chuang and CC Wu contributed equally to this work as corresponding authors.

**Supplemental Material table of contents**

**Supplemental Table 1.**

Comparison of the characteristics of study cohort and the 2017 nationwide registry in Taiwan

**Supplemental Table 2.**

Blood pressure variability metrics of the study participants

**Supplemental Table 3.**

Sensitivity analysis using time-varying BPV and covariates model in the Cox regression analysis for vascular access outcomes

**Supplemental Table 4.**

Sensitivity analysis using different metrics of baseline BPV in the Cox regression analysis for vascular access outcomes

**Supplemental Table 5.** Sensitivity analysis to evaluate the effect of systolic BPV on outcomes of vascular access after excluding thrombosis and stenosis events during hospitalization

**Supplemental Figure 1.**

Relationship between the number of blood pressure measurements and accuracy of variability assessment

**Supplemental Table 1.** Comparison of the characteristics of the study cohort and the 2017 nationwide registry in Taiwan

| **Factors** | **Study cohort N=1011** | **Nationwide registry N=82031** | | |  |
| --- | --- | --- | --- | --- | --- |
| Age, years | 66 (14) | 67 (12) | | |  |
| Male sex (%) | 52% | 52% | | |  |
| Diabetes (%) | 55% | 45% | | |  |
| Hypertension (%) | 90% | 84% | | |  |
| Coronary artery disease (%) | 35% | 37% | | |  |
| Heart failure (%) | 15% | 15% | | |  |
| Atrial fibrillation (%) | 6% | 6% | | |  |
| Cerebrovascular disease (%) | 9% | 12% | | |  |
| Hospital-based center (%) | 55% | 62% | | |  |
| Albumin, <3.5 g/L | 17% | 18% | | |  |
| Calcium X Phosphate, >55 | 24% | 19% | | |  |
| Hemoglobin, <10 g/dL | 28% | 40% | | |  |
| Values are mean (standard deviation) or proportions | | |  |  | |

**Supplemental Table 2.** Blood pressure variability metrics of the study participants

| **BPV parameters** | **SBP** | **DBP** |
| --- | --- | --- |
| Standard deviation, mmHg | 15.4 (4.5) | 8.4 (2.6) |
| Coefficient of variance, % | 10.9 (3.0) | 12.1 (0.38) |
| Variance independent of mean, % | 11.9 (3.0) | 18.3 (5.3) |
| Average real variability, mmHg | 15.4 (6.4) | 8.0 (2.8) |

Values expressed as mean (standard deviation)

Abbreviations: BPV, blood pressure variability; SBP, systolic blood pressure; DBP, diastolic blood pressure

**Supplemental Table 3.** Sensitivity analysis using time-varying BPV and covariates model in the Cox regression analysis for vascular access outcomes

| **Access type**  (Per SD increase) | **Stenosis** | | | | **Thrombosis** | | | |
| --- | --- | --- | --- | --- | --- | --- | --- | --- |
|  | **HR** | **LB** | **UB** | **P** | **HR** | **LB** | **UB** | **P** |
| Crude | 1.14 | 1.04 | 1.25 | 0.004 | 1.31 | 1.16 | 1.49 | <0.001 |
| Model 1 | 1.13 | 1.03 | 1.24 | 0.007 | 1.30 | 1.14 | 1.47 | <0.001 |
| Model 2 | 1.04 | 0.90 | 1.19 | 0.63 | 1.21 | 1.00 | 1.45 | 0.04 |
| Model 3 | 1.04 | 0.91 | 1.20 | 0.56 | 1.21 | 1.00 | 1.47 | 0.04 |

**Abbreviations:** CI, confidence interval; HR, hazard ratio; LB, lower bound; UB, upper bound

**Model 1:** Adjusted for age, sex, systolic blood pressure

**Model 2:** adjusted for factors with p value less than 0.1 in univariate analyses.

For analysis of thrombosis, including diabetes, coronary artery disease, peripheral artery disease, vascular access dysfunction, Charlson comorbidity index, urea clearance, intradialysis hypotension, institution, access types, and systolic blood pressure.

For analysis of stenosis, including Charlson comorbidity index, peripheral artery disease, vascular access dysfunction, urea clearance, intradialysis hypotension, institution, type of access, anti-hypertension medicine, and statin.

**Model 3:** All the factors in models 1 and 2

**Supplemental Table 4.** Sensitivity analysis using different metrics of baseline BPV in the Cox regression analysis for vascular access outcomes

| **BPV metrics**  (Per SD increase) | **Stenosis** | | | | **Thrombosis** | | | |
| --- | --- | --- | --- | --- | --- | --- | --- | --- |
|  | **HR** | **LB** | **UB** | **P** | **HR** | **LB** | **UB** | **P** |
| SD | 1.13 | 1.03 | 1.23 | 0.011 | 1.26 | 1.10 | 1.43 | 0.001 |
| CV | 1.17 | 1.08 | 1.28 | <0.001 | 1.36 | 1.21 | 1.52 | <0.001 |
| VIM | 1.52 | 1.11 | 2.07 | 0.009 | 2.45 | 1.59 | 3.80 | <0.001 |
| ARV | 1.1 | 1.02 | 1.20 | 0.02 | 1.17 | 1.04 | 1.31 | 0.009 |

Abbreviations: BPV, blood pressure variability; SD, standard deviation; HR, hazard ratio; LB, lower bound; UB, upper bound; CV, coefficient of variation; VIM, variance independent of mean; ARV, average real variability

**Supplemental Table 5.** Sensitivity analysis to evaluate the effect of systolic BPV on outcomes of vascular access after excluding thrombosis and stenosis events during hospitalization

| **Access type** | **Thrombosis** | | | | **Stenosis** | | | |
| --- | --- | --- | --- | --- | --- | --- | --- | --- |
|  | **HR** | **LB** | **UB** | **P** | **HR** | **LB** | **UB** | **P** |
| Crude | 1.14 | 1.05 | 1.27 | 0.002 | 1.32 | 1.18 | 1.49 | <0.001 |
| Model 1 | 1.14 | 1.04 | 1.24 | 0.004 | 1.31 | 1.16 | 1.48 | <0.001 |
| Model 2 | 1.06 | 0.97 | 1.16 | 0.23 | 1.23 | 1.08 | 1.40 | 0.002 |
| Model 3 | 1.06 | 0.97 | 1.16 | 0.19 | 1.24 | 1.08 | 1.41 | 0.001 |

**Abbreviations:** CI, confidence interval; HR, hazard ratio; LB, lower bound; UB, upper bound

**Model 1:** Adjusted for age, sex, systolic blood pressure

**Model 2:** adjusted for factors with p value less than 0.1 in univariate analyses.

For analysis of thrombosis, including diabetes, coronary artery disease, peripheral artery disease, vascular access dysfunction, Charlson comorbidity index, urea clearance, intradialysis hypotension, institution, access types, and systolic blood pressure.

For analysis of stenosis, including Charlson comorbidity index, peripheral artery disease, vascular access dysfunction, urea clearance, intradialysis hypotension, institution, type of access, anti-hypertension medicine, and statin.

**Model 3:** All the factors in models 1 and 2

**Supplemental Figure 1.** Relationship between the number of blood pressure measurements and accuracy of variability assessment


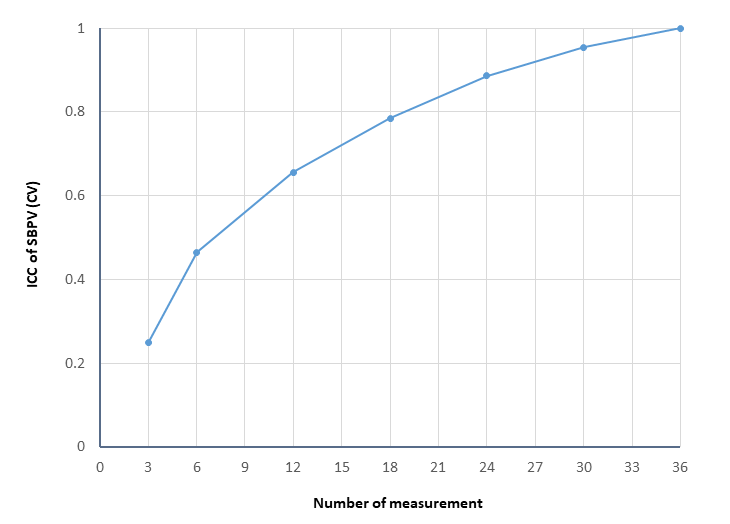


| **Number** | **Mean** | **Delta** | **r** | **P** | **ICC** | **LL** | **UL** | **P** | **Quality** |
| --- | --- | --- | --- | --- | --- | --- | --- | --- | --- |
| 36 | 0.11 | REF | REF | REF | REF | REF | REF | REF |  |
| 30 | 0.11 | 0.002 | 0.96 | <0.0001 | 0.96 | 0.95 | 0.96 | <0.0001 | Excellent |
| 24 | 0.11 | 0.003 | 0.89 | <0.0001 | 0.89 | 0.87 | 0.90 | <0.0001 | Good |
| 18 | 0.10 | 0.005 | 0.79 | <0.0001 | 0.79 | 0.76 | 0.81 | <0.0001 | Good |
| 12 | 0.10 | 0.007 | 0.67 | <0.0001 | 0.66 | 0.62 | 0.69 | <0.0001 | Moderate |
| 6 | 0.96 | 0.01 | 0.50 | <0.0001 | 0.47 | 0.41 | 0.51 | <0.0001 | Moderate |
| 3 | 0.88 | 0.02 | 0.30 | <0.0001 | 0.25 | 0.19 | 0.31 | <0.0001 | Poor |

Abbreviations: ICC, intraclass correlation coefficient; SBPV, systolic blood pressure variability; CV, coefficient of variance; LL, lower limit; UL, upper limit
